# Editorial: Advanced Silica Nanomaterials for Drug Delivery

**DOI:** 10.3389/fchem.2021.677647

**Published:** 2021-04-20

**Authors:** Xijian Liu, Yanyan Jiang, Pingqiang Cai, Ruixia Gao

**Affiliations:** ^1^College of Chemistry and Chemical Engineering, Shanghai University of Engineering Science, Shanghai, China; ^2^School of Materials Science and Engineering, Shandong University, Jinan, China; ^3^School of Materials Science and Engineering, Nanyang Technological University, Singapore, Singapore; ^4^School of Chemistry, Xi'an Jiaotong University, Xi'an, China

**Keywords:** drug delivery, silica, therapy, nanomaterials, theranostic agent

Chemotherapy is a commonly used clinical treatment, but it is limited by poor selectivity, high systemic toxicity, and multidrug resistance. To improve the therapeutic efficacy and optimize traditional chemotherapy, nanocarriers have been developed for drug delivery. In recent years, mesoporous silica nanoparticles (MSNs) have become exciting nanocarriers for targeted and controlled drug/protein/gene delivery due to their excellent stability, good biocompatibility, large surface and cavity volumes, tunable porosity, and facile modification. In addition, the silica can not only coat on the surface of nanoparticles to enhance their biocompatibility and stability but also may provide the substrate for functional nanoparticles growing on their surface to reach multifunction. When combining drug delivery with other treatments based on silica nanoparticles, they exhibit synergistic effects and achieve imaging guided therapy, significantly improving therapeutic efficiency.

This Research Topic invited researchers to contribute studies on recent advances in silica nanomaterials for drug delivery that enable a better understanding of the role of silica nanoparticles as a theranostic agent. We have collected four original research articles and one review, which highlight several emerging trends of silica nanomaterials.

Gao et al. provide an overview recent developments in MSNs drug delivery systems (DDS) for various chemotherapeutic combination anti-tumor treatments, including photothermal therapy, photodynamics therapy, gene therapy, immunotherapy, sonodynamic therapy, magnetic hyperthermia therapy, chemodynamic therapy, and cancer starvation therapy. The study examines the characteristics of each nanomaterial and the synergistic advantages of combined therapies are demonstrated. Though several challenges remain before clinical use, chemotherapy-based MSN DDS has a bright adaptable future and great potential for clinical translation. Paramonov et al. utilize an in-house established bioassay to deduce the genuine input of MSN-anchored peptides in the net receptor activation, thus verifying MSN targeted capability in protein-depleted and serum-enriched media. Mei et al. show how porous COS@SiO_2_ (Chitosan oligosaccharides, COS) nanocomposites could enable sustained release of COSs slowly with pH response and maintain a higher drug concentration. The porous COS@SiO_2_ nanocomposites activated the Nrf2 signaling pathway to inhibit oxidative stress, reduce the expression of NF-κB and the NLRP3 inflammasome, and decrease the release of inflammatory factors, thus blocking the systemic inflammatory response and ultimately ameliorating SAP and associated lung injury. Ma et al. developed Cu-containing mesoporous silica nanosphere-modified β-tricalcium phosphate (Cu-MSN-TCP) scaffolds. The Cu-MSN-TCP scaffolds can completely eradicate residual bone tumor cells and simultaneously heal large bone defects, which may provide a novel and effective strategy for bone tumor therapy. Fu et al. reported an NIR light-triggered drug delivery system [CuS@mSiO_2_-PEG nanoparticles loading a thrombolytic drug (urokinase plasminogen activators, uPA)]. These CuS@mSiO_2_-PEG nanoparticles exhibited a high photothermal conversion efficiency of 52.8% and a drug loading content of 8.2%. Drug release could be triggered by NIR irradiation, and the CuS@mSiO_2_-PEG/uPA had excellent thrombolytic ability under the irradiation of an 808 nm laser, showing a combined therapy for thrombolysis.

In summary, this Research Topic covers important developments in the use of MSNs as drug carriers, providing a better understanding of the role of MSNs in enhancing the therapeutic effect. MSNs have the advantages of large pore volume, controllable fabrication, easy functionalization, and high biocompatibility, which make them robust nanoplatforms for diagnosis and treatment. Thus, MSNs-based materials have great potential as versatile nanoplatforms for diagnosis and treatment in biomedical applications.

## Author Contributions

XL wrote the editorial, which was revised, proofed, and accepted by all the authors.

## Conflict of Interest

The authors declare that the research was conducted in the absence of any commercial or financial relationships that could be construed as a potential conflict of interest.

